# Bridging neurobiological mechanisms and translational applications in cognitive science: inspirations from the MCCS-NYUAD meeting 2025

**DOI:** 10.1186/s13041-026-01281-7

**Published:** 2026-02-28

**Authors:** Mohammad Nami, Dan Ohtan Wang, MCCS-NYUAD Panel, Ted Abel, Sourav Banerjee, Tim Bredy, Dipesh Chaudhury, Paul Frankland, Yasunori Hayashi, Hajime Hirase, Kaoru Inokuchi, Bong-Kiun Kaang, Satoshi Kida, Mazen Kheirbek, Kwok-on Lai, Yong-Seok Lee, David Melcher, Mohammad Nami, Kobi Rosenblum, Bas Rokers, Jennifer Raymond, Mohammed Shaker, Saji Kumar Sreedharan, Dan Ohtan Wang, Ki-Jun Yoon

**Affiliations:** 1 Cognitive Neuroscience Unit, School of Health Sciences and Psychology, Canadian University Dubai (CUD), Dubai, UAE; 2 RNA-MIND Lab, Biology Program, New York University Abu Dhabi (NYUAD), Abu Dhabi, UAE; 3 Center for Brain and Health, New York University Abu Dhabi (NYUAD), Abu Dhabi, UAE; 4 University of Iowa, Iowa City, IA USA; 5 National Brain Research Centre of India, Gurugram, India; 6 The University of Queensland, Brisbane, Australia; 7 New York University Abu Dhabi, Abu Dhabi, UAE; 8 Hospital for Sick Children Toronto, Toronto, Canada; 9 Kyoto University, Kyoto, Japan; 10 University of Copenhagen, Copenhagen, Denmark; 11 University of Toyama, Toyama City, Japan; 12 Institute for Basic Science, Daejeon, South Korea; 13 University of Tokyo, Tokyo, Japan; 14 University of California San Francisco, San Francisco, CA USA; 15 City University of Hong Kong, Hong Kong, China; 16 Seoul National University, Seoul, South Korea; 17 Canadian University Dubai, Dubai, UAE; 18 University of Haifa, Haifa, Israel; 19 Stanford University, Stanford, CA USA; 20 Hamad Bin Khalifa University, Doha, Qatar; 21 National University of Singapore, Singapore, Singapore; 22 Korea Advanced Institute of Science and Technology, Daejeon, South Korea

**Keywords:** Neuroplasticity, Epigenetic regulation, Precision neurocognitive medicine, Synaptic memory consolidation, Neuroimmune interactions

## Abstract

The Molecular and Cellular Cognition Society (MCCS) Meeting (NYU Abu Dhabi, February 17-18th, 2025) brought together leading experts in neuroscience to present breakthroughs addressing the molecular and neuronal mechanisms underlying cognition, emotion, and behavior. This review is inspired by the meeting, which emphasized emerging molecular and cellular mechanisms including epigenetic regulation of memory, dynamic engram synapse formation, synaptic epitranscriptomics, metaplasticity, and metabolomic-neuroimmune interactions. Learning and cognition have increasingly become focal points within broader advances in neuroimaging innovations, high-throughput molecular diagnostics, and computational modeling geared toward precision neurodiagnostics and personalized neurocognitive therapeutics. The meeting also scrutinized how stress, circadian rhythm disruption, and neuroinflammation converge to shape cognitive resilience and dictate dysregulated attention and learning mechanisms underlying cognitive dysfunction. Such conditions span neurodevelopmental, neuropsychiatric, and neurodegenerative disorders. Collectively, the studies highlighted how experience-dependent synaptic and circuit-level changes influence cognition, sensory integration, and motor output. Further discussions addressed the translational implications of these findings, including their potential to advance neurotechnologies such as targeted neuromodulation, pharmacogenomic interventions, and AI-based biomarker discovery. Drawing on the scientific discussions at the MCCS-NYUAD meeting, we synthesize a research roadmap for the future of precision neurocognitive medicine by integrating molecular cognition with clinical neuroscience. Future research priorities include bridging gaps in molecular biomarkers of neurocognitive aging and leveraging AI-driven neurodiagnostics and large-scale biological data analytics. Overall, the meeting laid the groundwork for a shift in neuroscience toward linking mechanistic understanding with clinical relevance to enhance cognitive health and develop targeted neurotherapeutic approaches.

## Introduction

As precision neurodiagnostics and personalized neurocognitive therapeutics advance, it has become increasingly clear that a fundamental understanding of how molecular and cellular mechanisms give rise to cognitive functions such as learning, memory, and emotion has significant implications. These scientific discoveries drive innovative approaches in functional neuroimaging, pharmacogenomics, neuromodulatory interventions, and AI-based biomarker analysis, collectively reshaping the diagnosis of cognitive deficits, the prediction of disease trajectories, and the range of clinical interventions. The prediction, prevention, precision, and participatory (P4) approach in neurocognitive and psychiatric disorders is expected to substantially reduce the economic burden of such predicaments globally, as outlined in the N20 summit (Neuroscience 20) [1].

In the same vein, the Molecular and Cellular Cognition Society Meeting (February 17-18th, 2025) at NYU Abu Dhabi (MCCS-NYUAD) gathered the field’s experts in neuroscience, cognitive science, and neurotechnology to examine the complex molecular, genetic, and neurophysiological mechanisms that lead to cognition, emotion, and behavior. The meeting focused on the leading edge of innovation in molecular neuroscience, neuroplasticity, and computational modeling to translate fundamental research principles into precision neurodiagnostics and neurocognitive therapeutics.

Right at the beginning of the forum, it was clarified that memory is not a fixed endpoint function but rather an interplay of dynamic and integrated molecular and neuronal processes. Further discussions covered epigenetic landscapes in memory, synaptic plasticity, engram synapse stabilization, the role of long-coding RNAs (lncRNAs) in cognition, and synaptic epitranscriptomics. The studies in these domains have provided new insights into the ways cellular changes enable us to formulate solutions to endure cognitive challenges. Other research domains highlighted were metabolomics and neuroimmune interactions, specifically how stress, circadian disruptions, and inflammatory responses affect cognitive health and neurodevelopment. Besides bottom-up lessons from foundational neuroscience, the forum highlighted translational applications in precision medicine, including novel diagnostics and therapy strategies for neurodevelopmental, neuropsychiatric, and neurodegenerative disorders.

**Fig. 1:**
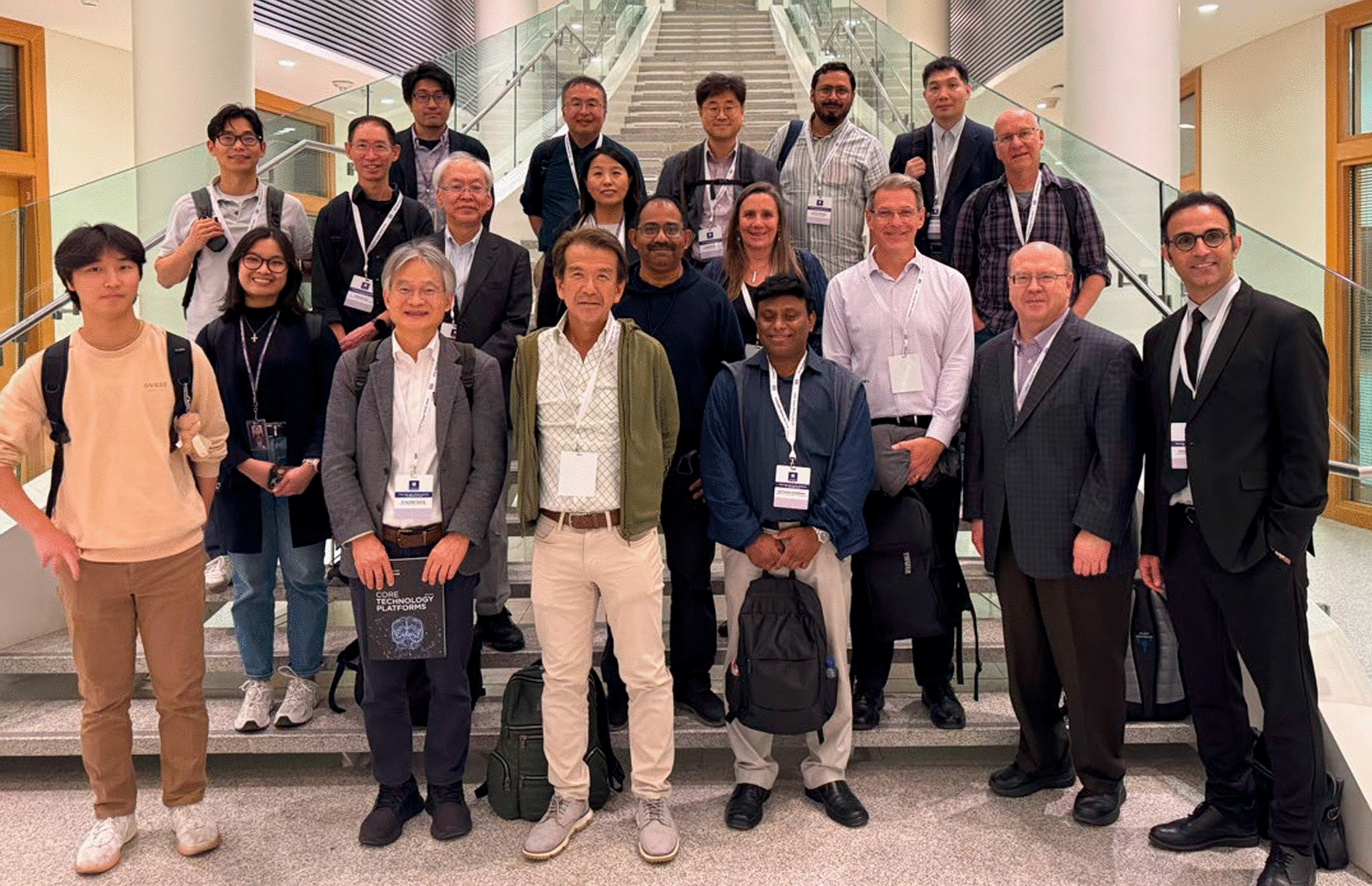
Experts’ panel at the MCCS-NYUAD Meeting (February 17-18th, 2025). Alphabetically-Ted Abel, Sourav Banerjee, Tim Bredy, Dipesh Chaudhury, Paul Frankland, Yasunori Hayashi, Hajime Hirase, Kaoru Inokuchi, Bong-Kiun Kaang, Satoshi Kida, Mazen Kheirbek, Kwok-on Lai, Yong-Seok Lee, David Melcher, Mohammad Nami, Kobi Rosenblum, Jennifer Raymond, Mohammed Shaker, Saji Kumar Sreedharan, Ohtan Wang, and Ki-Jun Yoon

The two-day discussions underscored emerging personalized neurocognitive medicine by integrating discoveries of molecular, cellular, and systems-level neuroscience with innovative approaches to functional neuroimaging, pharmacogenomics, neuromodulatory interventions, and AI-based biomarker analysis merging in a demonstration of how data-based neuroscience can inform clinical interventions in the future (Fig. 1).

## Molecular and epigenetic mechanisms in memory and cognition

### Epigenetic mechanisms of memory storage, extinction, and gene-environment interactions

Chromatin-based epigenetic regulation, mediated by dynamic chemical modifications of histone proteins and DNA, influences transcriptional activity of genes that support synaptic plasticity and neuronal function underlying long-term memory formation and maintenance. Studies of epigenetic regulation of memory in cognition-related regions, such as the hippocampus and prefrontal cortex, have provided deeper insight into how environmental factors interact with molecular mechanisms during memory development. These findings underscore the view that memory is not an endpoint product but a continuation of molecular events unfolding along a branching trajectory over time [2].

For example, cAMP and Ca^2+^ serve as major signaling molecules through which synaptic activity is communicated to chromatin, driving epigenetic changes via phosphorylation of transcription factors (e.g. CREB) that recruit histone acetyltransferases (HATs) to activate specific transcriptional programs. Context-specific neurochemical signals (i.e., dopamine, acetylcholine) further act to tag or consolidate sensory experience into long-term memory [3]. These memories outlive the initial transient stimulus and modify brain circuits to guide future adaptive responses to the environment. Thus, epigenetically regulated, activity-dependent gene expression underlies gene-environment interactions, the disruption of which has been linked to addiction, stress-related disorders, neurodegeneration, post-traumatic stress disorder, and major depressive disorder [4–6].

The dynamic nature of epigenetic modifications raises the possibility of therapeutically tuning enzyme activities to promote memory recovery in aging and disease. Moreover, it opens avenues for precise, targeted therapies for psychiatric disorders. Together, these advances represent important steps toward translational precision medicine, in which epigenetic landscape-targeted interventions may enhance cognition and ameliorate memory-related pathologies, marking the dawn of a new era of tailored therapeutics for cognitive disorders [6].

### Synaptic epitranscriptomics

Unlike DNA, RNA molecules are distributed throughout the cell, including distal compartments of neurons and glia such as synapses and growth cones [7]. Epitranscriptomics, a burgeoning field that collectively refers to the study of diverse chemical modifications of RNAs, has emerged as a fundamental molecular regulatory mechanism across all domains of life. Synaptic epitranscriptomics, referring to the regulation of synaptic function through local RNA chemical modifications, has gained increasing attention for its contribution to synaptic function and plasticity underlying neural adaptability, memory encoding, and information processing.

During the MCCS-NYUAD meeting, it was emphasized how RNA modifications, including N6-methyladenosine (m6A) methylation, dynamically regulate local translation and structural plasticity within neuronal circuits [8]. These enzyme-mediated chemical marks on the RNA molecules govern the stability, transport, and translation of synaptic mRNAs, enabling rapid and spatially restricted adaptation of protein synthesis to the demands of ongoing cognitive processes. Importantly, activity-dependent epitranscriptomic regulation within dendritic spines not only fine-tunes synaptic RNA composition and translational status but also modulates synaptic strength through regulated local translation. This mechanism circumvents slower genetic and chromatin-based epigenetic regulations, allowing rapid responses during neurodevelopment, injury repair, and stress adaptation [8–12].

The role of cytoplasmic reader proteins of m6A in trafficking mRNAs into developing axons was discussed. In addition to marking specific mRNAs, a new model was proposed in which m6A-mediated translation control of the RNA-binding protein APC regulates the axonal transport and local translation of *Actb* mRNA, thereby supporting axon development [13]. Advances in neurodiagnostics and molecular imaging have shed new light on epitranscriptomic mechanisms involved in synaptic homeostasis and cognitive resilience. Accordingly, discussions at the meeting focused on how RNA methyltransferases and demethylases coordinate the post-transcriptional landscape at engram synapses, thereby regulating synaptic efficacy and memory persistence. Aberrant patterns of synaptic RNA modifications have been linked to neurodevelopmental disorders and cognitive impairment, positioning epitranscriptomic signatures as potential diagnostic biomarkers [10, 14].

Looking forward, the combination of direct RNA sequencing, molecular neuroimaging, and machine learning-based biomarker analyses may accelerate the development of tailored interventions that leverage epitranscriptomics to enhance synaptic plasticity and cognitive function.

### Synapse-enriched long non-coding RNAs in memory

Recent studies presented at the MCCS-NYUAD meeting highlighted the importance of synaptically localized long non-coding RNAs (lncRNAs) in memory formation and cognitive resilience. Unlike protein coding RNAs, lncRNAs, in both linear and circular forms, modulate gene expression through epigenetic, transcriptional, and post-transcriptional mechanisms that influence synaptic plasticity and neuronal circuit stability. Dynamic lncRNA expression has been documented in brain regions critical for learning, memory, and perception (e.g., the hippocampus and prefrontal cortex), contributing to experience-driven plasticity in which synaptic architecture adapts to environmental stimuli.

Activity-dependent lncRNAs have been identified to serve as synapse-specific scaffolds that facilitate the local translation of synaptic proteins necessary for memory consolidation and extinction [15–18]. Furthermore, synapse-enriched lncRNAs regulate neurotransmitter receptor composition and drive synaptic remodeling underlying long-term memory consolidation [15, 17]. As the field advances, integrating lncRNA profiling with sophisticated neuroimaging approaches is likely to open new diagnostic and therapeutic avenues for enhancing synaptic resilience and preventing or mitigating memory-related deficits.

## Neuronal network plasticity and dynamics

### Engram synapse and memory consolidation

During memory consolidation, persistent synaptic adaptations arising from synaptic plasticity enable the long-term storage and retrieval of learned information, thereby forming an “engram” embedded within synaptic connections [19]. The salience and stability of memory traces arise from the intricate and highly non-linear molecular circuitry that governs engram synapse formation as well as synaptic (and anti-synaptic) plasticity and structural remodeling [20–22].

Using highly bright dual-eGRASP (enhanced green fluorescent protein reconstitution across synaptic partners) technology to label functional synapses between defined neuronal populations (e.g. engram cells), specific connections between engram and non-engram neurons can be independently visualized within the same animals, rendering direct visualization of functional connectivity [22]. Notably, engram synapse formation depends on de novo protein synthesis, as anisomycin injections abolish the plasticity, linking the engram synapse to RNA biology-mediated molecular mechanisms in the previous section [23, 24]. Visualization of engram synapses further reveals the formation of clustered synaptic engrams, which may contribute to the encoding of episodic memories [25].

A logical extension of memory consolidation is “forgetting”, which arises from reduced retrievability of stored information, initially through loss of details leading to a “gist”-based memories, and ultimately to more “schematized" representations [26, 27]. Receptor trafficking, cytoskeleton remodeling, and neuromodulatory signaling, particularly involving dopamine and acetylcholine, are proposed as key regulatory molecular mechanisms of reversing synaptic weight during “forgetting” [26]. Discussions at the meeting further explored how engram synapse dynamics allow incoming sensory inputs to be flexibly segmented, enabling different memory components to acquire different binding strengths while remaining stably and coherently integrated into a retrievable cognitive-representation [19, 28].

Beyond synaptic strengthening, researchers are increasingly focused on the molecular and epigenetic changes in multiple brain cell types that accompany long-term memory consolidation. Over timescales of hours and days, histone modifications and DNA methylation contribute to the stabilization of synaptic changes, effectively consolidating memory traces [29–32]. In parallel, neuroimaging strategies (e.g., PET scanning and molecular markers) have begun to provide insights into how engram synapses are reshaped by environmental inputs and provide potential diagnostic readouts for cognitive resilience [33].

Collectively, these advances open new avenues for precision-medicine approaches that target engram synapses to enhance cognitive function and to develop therapeutic strategies for memory-related disorders [21, 26, 34, 35]. Drawing on tools from molecular biology, neuroimaging, and computational neuroscience, the field is coalescing around a common theory of how memory consolidation works and for manipulating engram synapse dynamics to improve learning, memory retention, and overall cognitive health.

### Location memory in anterior cingulate cortex (ACC)

The anterior cingulate cortex (ACC), which connects with both the limbic system and the cerebral cortex, is uniquely positioned to bridge emotional and cognitive functions. It plays critical roles in location memory, motivation, salience assessment, decision-making, and adaptive behaviors [36–44]. The ACC participates in the consolidation of spatial representation during sleep following hippocampus-dependent learning on the preceding day [45, 46]. Mechanisms of synaptic plasticity, including modulation by neurotransmitters such as dopamine and acetylcholine, dynamically reconfigure spatial encoding within the ACC, enabling the integration of environmental cues with higher-order cognitive processes such as attention and conflict monitoring. Furthermore, interactions between sensory segmentation and spatial cognition within the ACC facilitate effective memory consolidation, thereby supporting goal-directed navigation and decision-making [46, 47].

Advances in neuroimaging and molecular diagnostics have provided additional insight into the neurobiological basis of ACC-dependent location memory [48]. Functional MRI and PET studies suggest that differences between neurotransmitter receptor distribution within the ACC correlate with spatial learning efficiency [49, 50]. At the molecular level, synaptic reorganization and the epigenetic landscape in the ACC appear to determine the durability of location-specific memory traces. Given its central role in cognitive flexibility and spatial awareness, the ACC represents a promising target for neuromodulatory interventions and may offer therapeutic opportunities to enhance memory function in neurodegenerative and psychiatric disorders [51].

Future studies integrating electrophysiology, molecular profiling, and computational modeling will be crucial for unifying these findings and to elucidate how the ACC encodes and retrieves location-specific memories, paving the way for precision treatments to improve cognition.

### Hippocampal CA2-to-CA1 metaplastic switch

The hippocampal CA2-to-CA1 metaplastic switch is a key regulator that modulates synaptic plasticity, and consequently, episodic memory encoding and retrieval. The meeting highlighted the relatively understudied hippocampal subregion CA2. CA2 supports and perhaps modulates CA1-dependent memory processes; whereas CA3 serves as a hub for auto-associative retrieval, CA2 exhibits distinct synaptic characteristics to gate the threshold for long-term potentiation (LTP) in CA1, thereby acting as a metaplasticity gatekeeper [52].

This dynamic switching mechanism is modulated by NMDA receptor activity, calcium influx, and neuromodulatory signals, including vasopressin and dopamine. Together these factors allow CA1 synapses to switch in and out of distinct functional states, enabling the synapses to respond appropriately to continuously changing cognitive demands while preventing synaptic saturation or depression. Such regulation is essential for memory flexibility and for protecting synaptic dysfunction associated with cognitive impairment.

Recent advances in molecular neuroimaging and electrophysiological recording have further shed more light on the biophysical basis for this CA2-to-CA1 metaplastic transition [53, 54]. The role of epigenetic modifications and synaptic RNA regulation in shaping plasticity within these hippocampal circuits were emphasized [54, 55]. In particular, alterations in synaptic tagging and capture processes within CA2 determine whether activity-dependent metaplastic are stabilized in CA1, thereby influencing learning efficiency and spatial navigation [56, 57]. Dysregulation of this metaplastic switch has been linked to neurodevelopmental disorders and memory deficits, making CA2 a promising therapeutic target for precision neuromodulatory interventions.

Future studies integrating functional imaging, optogenetics, and computational models are likely to yield deeper insights into CA2’s role in orchestrating hippocampal-wide plasticity, ultimately informing the development of innovative strategies for cognitive enhancement and the treatment of memory disorders.

### Cerebellar metaplasticity

Unique among its functions, cerebellar metaplasticity, the ability of the cerebellum to dynamically regulate thresholds of synaptic plasticity, has only recently emerged as a key mechanism underlying adaptive motor control and cognitive flexibility [58].

Key takeaway from the MCCS-NYUAD meeting highlighted the dynamic role of the cerebellum, traditionally associated with motor coordination, in higher-order cognitive processes such as learning and memory. Studies emphasized the role of calcium-dependent signaling cascades and neurotransmitter kinetics, particularly involving glutamate and GABA, as critical regulators that fine-tune cerebellar synaptic plasticity across learning episodes [59, 60]. Within Purkinje cell networks and deep cerebellar nuclei, metaplasticity allows the cerebellum to adjust synaptic strength to ongoing neural activity and mediate motor fine-tuning and error correction. While synaptic plasticity is essential for learning, metaplastic regulation preserves circuit stability by preventing excessive potentiation or depression that could otherwise disrupt coordinated movement and information processing [58, 60].

More recently, neuroimaging and electrophysiological approaches have revealed that cerebellar metaplasticity also supports cognitive resilience and efficient lifelong learning. These findings imply that cerebellar synaptic modifications not only optimize motor output, but also interact with prefrontal and parietal networks, shaping attention and our executive abilities [61]. Furthermore, molecular analyses have begun to elucidate the contributions of epigenetic regulation and synaptic RNA homeostasis to cerebellar metaplasticity, pointing toward potential therapeutic mechanisms for movement disorders and cognitive impairments. Future studies integrating functional neuroimaging, molecular profiling techniques, and neuromodulatory interventions are poised to be informative of how cerebellar metaplasticity promotes neuroadaptive processes, paving the way for precision medicine-based strategies for neurorehabilitation and cognitive enhancement [60, 62].

### Neural circuits for social memory consolidation

Social memory, a higher-order cognitive function, relies on a distributed neural network that integrates emotional, cognitive, and sensory information, enabling progressive recognition and recall of individuals over time. Studies discussed at the MCCS-NYUAD meeting indicate that the hippocampus, amygdala, and prefrontal cortex are critically involved in the encoding and stabilization of social memories [63, 64].

The CA2 subregion of the hippocampus, which is relatively resistant to plasticity saturation, has emerged as a key relay for transmitting social recognition signals to CA1, supporting the long-term representations of social memory [63, 65]. Neuromodulatory systems, including oxytocin- and vasopressin-mediated signaling, regulate synaptic efficacy within these circuits and enhance the salience and consolidation of social interactions. The amygdala, especially the basolateral nucleus, refines social memory by associating emotional valence with specific individuals, allowing reward outcomes to be integrated into social memory and salient interactions are preferentially stored [66, 67].

Recent neuroimaging and molecular studies have further illuminated the synaptic and epigenetic mechanisms that support social memory consolidation [68–70]. Furthermore, dopaminergic projections from the ventral tegmental area (VTA) to the hippocampus and medial prefrontal cortex reinforce social learning and remodel neural circuitry in a context- and experience-dependent manner [71–73]. Dysregulation of these pathways has been linked to neurodevelopmental and psychiatric disorders, including autism spectrum disorder and schizophrenia, fostering the development of targeted therapeutic approaches [74, 75]. As such, future studies integrating optogenetics, in vivo imaging, and molecular profiling are likely to yield novel strategies to enhance social memory and to ameliorate deficits in social cognitive function.

### The “idling” brain in cognition (resting-state neural activity and cognitive flexibility)

Idling brain activity refers to the offline neural dynamics that emerge when subjects are not engaged in any explicit task [76, 77]. Such spontaneous neural activity during resting state or sleep is critical to cognition, supporting the maintenance of functional connectivity and enabling the brain to integrate past experiences and prepare for future demands [77, 78].

Even in the absence of an external task, the brain performs highly nuanced functions that rely on spontaneous neural oscillations and network coordination. Fluctuations in activity within large-scale networks, particularly the default mode network (DMN), a collection of interconnected regions, including medial prefrontal cortex, posterior cingulate cortex, and the hippocampus, has been shown to support internally directed processes such as self-reflection, memory consolidation, and future planning [77, 79, 80]. Low-frequency oscillations of neural activities during idling states, including sleep, reinforce synaptic homeostasis while preparing the brain to efficiently process incoming external stimuli [79, 81].

Resting-state dynamics also serve as a neural substrate for cognitive flexibility which depends on efficient transitions between large-scale networks. This enables the translation of internally driven thoughts into task-relevant behavior, improving performance on tasks that require flexible thinking [78, 82]. Impairments in cognitive flexibility are associated with neurological disorders such as depression, anxiety disorders, obsessive–compulsive disorder (OCD), schizophrenia and post-traumatic stress disorder (PTSD). Thus, targeting resting-state aberrance may present a novel angle for disease intervention [83].

The meeting highlighted the recent evidence linking cognitive efficiency and neuroplasticity to idling-state brain activity, as observed through functional neural imaging and electrophysiological experiments [80, 84]. Variations in resting-state connectivity have been shown to predict individual differences in learning capacity, working memory, and problem-solving ability [78, 79]. Neurotransmitters (e.g., dopamine and acetylcholine) shape spontaneous neural activity patterns, influencing memory encoding and consolidation, and demonstrating how regulated neurotransmitter release can prime the brain for advantageous cognitive engagement [85].

Future work combining neuroimaging, computational modeling, and neuromodulation strategies will facilitate a more nuanced understanding of idling-state dynamics and open new avenues for enhancing cognitive flexibility and mental health.

### Reorganization of hippocampal activity networks during associative learning

Studies presented at the meeting examined population dynamics in the hippocampus underlying associative learning [86]. The hippocampal formation displays a sophisticated functional gradient where the dentate gyrus (DG) acts as a computational hub that disambiguates cortical sensory representations using inputs from the lateral entorhinal cortex (LEC) [87].

While the dorsal CA1 (dCA1) robustly represents stimulus identities regardless of their behavioral relevance, representations in the ventral CA1 (vCA1) emerge primarily through learning and the acquisition of predictive value. This functional division suggests that the dorsal axis is biased toward mapping precise external variables, whereas the ventral axis emphasizes internal states and imbues environmental cues with meaning [88].

Neural ensembles within the vCA1 robustly encode stimulus identity, modality, and intensity, but contrary to long-standing hypotheses, they do not represent the generalized valence (positive or negative quality) of these stimuli [86]. These internal representations of identity remain stable across days even when the emotional outcome of a stimulus is radically altered, such as when an appetitive reward is switched to an aversive outcome through reversal learning or conditioned taste aversion. This stable encoding logic is supported by a diverse vCA1 architecture where the majority of neurons project to single downstream targets, though a specific population broadcasts information to multiple regions simultaneously to coordinate emotional responses [89].

These insights provide a framework for how the brain integrates sensory, affective, and contextual learning into a coherent cognitive map. DG facilitates contextual recall by using sensory cues to drive pattern completion, a process where neural classification accuracy directly correlates with an individual’s ability to discriminate between different environments. Meanwhile, vCA1 serves as a permissive gatekeeper that identifies salient events and directs them to extended affective networks, such as the amygdala, where emotional values are ultimately integrated. Understanding how the hippocampus separates "what" an event is from "how good or bad" it is offers significant potential for developing circuit-based biomarkers to treat psychiatric and neurodegenerative disorders where these integrated learning processes become dysfunctional [90].

As neuroimaging, optogenetic, and molecular profiling approaches have become increasingly sophisticated, the way that hippocampal circuits dynamically interact with amygdala and prefrontal cortex to build meaningful representations of the environment has come into sharper focus. Future studies incorporating real-time electrophysiological recording with computational modeling and targeted neuromodulation may lead to novel approaches for intervention aimed at reshaping hippocampal reward-aversion circuits, which could serve as powerful therapeutics for mood and cognitive disorders.

## Neuroimmune, stress, and metabolomic interactions

### Association between stress, circadian rhythms, and sleep

Stress, circadian rhythms, and sleep-and their interactions-are key determinants of cognitive health and emotional resilience.

One report presented at the MCCS-NYUAD meeting demonstrated how chronic stress interferes with the body’s circadian regulatory mechanisms, leading to dysregulated sleep and impaired cognitive processing [91]. Projections from the lateral habenula (LHb) to the dorsal raphe nucleus (DRN), which normally regulate mood and circadian rhythms, were shown to be affected in mice susceptible to chronic social stress [92]. Moreover, while susceptible mice exhibited daily locomotor rhythms similar to those of stress-resilient and stress-naive mice, they were slower to re-entrain to a shifted light–dark cycle. These findings indicate that stress impairs the ability of the suprachiasmatic nucleus (SCN), the brain’s master circadian pacemaker, to adapt to environmental light cues.

Such interference likely disrupts clock rhythms and sleep–wake cycles, leading to fragmented sleep. These disturbances may compromise hippocampal-dependent memory consolidation and executive function, increasing vulnerability to anxiety and mood disorders [93–95]. Circadian misalignment also alters neurotransmitter systems, including serotonin and GABA, hampering the brain’s capacity to transition efficiently between states of alertness and restorative sleep [96, 97].

Moreover, alterations in slow-wave sleep (SWS) and rapid eye movement (REM) cycles, critical periods for synaptic remodeling and emotional processing, have been linked to cognitive impairments and neuropsychiatric disorders [93, 98, 99].

Therapeutic strategies such as light therapy, chronopharmacology, and targeted neuromodulation have demonstrated potential in restoring circadian stability and mitigating the cognitive and emotional consequences of stress-related sleep disturbances [100]. Future work with longitudinal sleep monitoring, molecular circadian profiling, and targeted interventions may advance precision medicine approaches aimed at optimizing sleep health and enhancing stress resilience.

### Anterior–posterior insula circuit and conditioned immune response

Conditioned immune response (CIR) allows a conditioned stimulus (e.g. a tastant) to become associated with an unconditioned stimulus, thereby activating or inhibiting the immune function. This process involves brain regions including insular cortex (IC), amygdala, and hypothalamus.

A recent study presented at the MCCS-NYUAD meeting indicated that bidirectional connectivity between the anterior and posterior insula cortex (aIC-pIC) is essential for retrieving CIR. The aIC and pIC differ in functionality: the aIC primarily processes interoceptive, emotional, and autonomic signals, whereas the pIC processes somatosensory and homeostatic signals [101]. Chemogenetic silencing of aIC-pIC projections disrupted both behavioral and immune responses, suggesting that immune responses can be a learned and reactivated experience shaped by aIC-pIC connectivity. Previously, the same group demonstrated that connectivity between neurons in different layers of the aIC and the basolateral amygdala (BLA) encodes the certainty of taste valence prediction. Notably, the excitability of aIC-BLA neurons is negatively correlated with outcome certainty [102].

Advances in immunological analysis have further elucidated the insular cortex’s role in modulating immune-brain crosstalk [103]. Moreover, aIC-pIC connectivity has been implicated in chronic pain, depression, and neurodegenerative disorders, all of which involve immune dysregulation [101]. Future studies integrating fMRI, neuroimmune profiling, and targeted neuromodulation may offer new therapeutic strategies for modulating insula-dependent immune responses, furnishing prospective targets for inflammatory and stress-related pathologies.

### Imaging tools for brain microcirculation studies

Monitoring blood circulation is essential for understanding cognitive functions because the vascular system serves as the primary life-support network for the brain, and its performance is directly linked to both healthy neural activity and the progression of neurological disorders. To monitor blood circulation using fluorescence in animals, the traditional method is to repeatedly inject synthetic dyes (such as indocyanine green or FITC-dextran), which is invasive, causes animal stress, and carries the risk of immune responses.

At the meeting, a genetically encoded labeling system designed for the long-term, stable visualization of plasma and microcirculation in animal models was presented. It utilizes the liver’s natural capacity to synthesize albumin to create a "permanent" fluorescent signal within the bloodstream [104]. A variety of recombinant fluorescent protein-tagged albumin markers (such as Alb-mNeonGreen, Alb-mScarlet, or Alb-mCarmine) were introduced. They can be delivered via a single injection of adeno-associated viral (AAV) vectors or implemented through CRISPR/Cas9 genome editing to ensure the fluorescent label is continuously produced by the liver and secreted into the circulation [105, 106]. Recent advancements have also introduced a dedicated transgenic mouse line (Alb-mSc-ST) that incorporates a "SpyTag" for modular attachment of various biosensors. The brightness of the dyes and the abundance of albumin in the blood stream allows the smallest vessels to be imaged in the microcirculation [106].

Stable labeling allows chronic imaging of the same vessels for over four months, allowing researchers to track vascular development, aging, and disease progression at high temporal resolution. Such imaging can be used to monitor behavior-related hyperemia and vascular plasticity after injury. When paired with the SpyTag-SpyCatcher system, it can act as a platform to monitor circulating pH, lactate, and potassium levels using specialized biosensors [106].

## Neurocognitive, sensory, and motor processing in health and disease

### White matter plasticity post-cataract surgery

Research presented at the meeting on congenital blindness and sight restoration has challenged traditional views regarding the "sensitive period" for visual development, which was historically thought to close around five to seven years of age. Pedersini et al. [107] demonstrates substantial neuroplasticity remains throughout adolescence, as patients aged 7 to 17 who underwent cataract surgery showed significant behavioral and structural improvements. While early-visual pathways, such as the optic tract and optic radiation, show limited structural change after this period, late-visual pathways exhibit remarkable surgery-related plasticity [107]. The reorganization of white matter after congenital cataract surgery demonstrates the brain’s ability to reorganize sensory pathways and sensory information processing after restoration of input.

Beyond structural white matter changes, cataract surgery initiates a broader process of visual neuroadaptation and functional recovery that impacts long-term health. For instance, resting-state fMRI analysis has shown that the brain must adapt to the specific optical properties of different intraocular lenses, such as multifocal lenses, which initially decrease visual cortical activity before it recovers and improves over several months [108]. Furthermore, this restoration of visual input and the resulting neural recovery have been linked to a significant slowing of cognitive decline in older adults, supporting the "cascade hypothesis" where improved sensory input reduces neurobiological atrophy. These neural and cognitive benefits are complemented by profound psychological relief, as successful surgery leads to a drastic reduction in symptoms of depression and generalized anxiety by restoring functional independence [109, 110].

Insights into these mechanisms could inform rehabilitation strategies, such as neurovisual training and neurostimulation methods [111] to maximize the white matter plasticity and potentially improve post-cataract surgical outcomes after the “sensitive period” of development, even in aging populations vulnerable to cognitive decline [109].

### Neural correlates of visual temporal resolution

How does a human brain parse a continuous stream of visual information into discrete perceptual units, a process known as temporal resolution? Understanding the neural correlates of visual temporal resolution for integration and segregation is crucial for understanding brain function and disorders. The evidence presented at the meeting demonstrates that while the neural oscillations, specifically in the alpha (8–12 Hz) and theta (4–7 Hz) bands, governs this process, aperiodic activity such as the "1/f" slope of the EEG spectrum is also functional relevant and affect the variability of visual temporal processing [112]. Understanding these mechanisms is crucial for characterizing how the brain maintains perceptual stability and why these processes are often disrupted in neurodevelopmental conditions like developmental dyslexia [113].

To investigate these mechanisms, researchers utilized a variety of neuroimaging and behavioral paradigms. A common behavioral tool is the two-flash fusion (TFF) task, where participants distinguish whether they see one or two sequential flashes, and the temporal integration/segregation (SegInt) task, which asks participants to either combine or separate visual elements. Methodologically, EEG and MEG were used to correlate resting-state or prestimulus brain activity with task performance. To establish causality, studies employed transcranial alternating current stimulation (tACS) to drive brain rhythms at specific frequencies and audiovisual entrainment to align internal oscillations with external sensory rhythms. Furthermore, source-level multivariate decoding in MEG allowed for the identification of specific cortical networks subserving these different sampling scales [114–117].

The findings demonstrate that visual temporal resolution is determined by both the speed and phase of ongoing rhythms. Faster alpha oscillations promote better temporal segregation, while the ongoing phase determines whether sequential stimuli are integrated into a single percept or parsed into two. MEG results reveal that rapid temporal sampling (TFF) is supported by a network involving early visual areas (V2) and V5/MT at alpha speeds, whereas slower spatio-temporal integration (AM) relies on the intraparietal sulcus (IPS) at theta speeds. Crucially, aperiodic neural noise also limits performance; a flatter EEG power spectrum (indicating an E/I imbalance toward excitation) predicts higher perceptual variability and inconsistent integration/segregation. In adults with developmental dyslexia, a synergy of impaired alpha modulation and altered neural noise leads to significant visual sampling deficits [113].

Future research built on such findings can move toward individualized clinical interventions and more complex, real-world applications. There is a clear need to refine tACS protocols by using individualized electric field modeling and custom-tailored frequencies to increase the reliability of neuromodulation [118]. Future work should also address how multiple coexisting rhythms interact during complex tasks, such as natural reading or object tracking, which likely require hierarchies of different temporal scales. Since binding deficits and "oscillopathies" are markers for disorders like schizophrenia, autism, and dyslexia, future studies could investigate whether targeted oscillatory training can improve perceptual resolution and cognitive outcomes in these populations.

### Studying childhood hypomyelination using iPSC-based organoids

The use of patient-specific iPSCs to study Hypomyelination with Brainstem and Spinal Cord Involvement and Leg Spasticity (HBSL) was discussed at the meeting. HBSL (OMIM: 615,281) is a rare, autosomal recessive, early-onset childhood leukodystrophy characterized by motor impairment, spasticity, nystagmus, and cerebellar ataxia [119]. No consensus exists for HBSL treatment. Recessive mutations in the DARS1 gene, which encodes cytosolic aspartyl-tRNA synthetase (AspRS), has been associated with HBSL. This enzyme charges transfer RNA (tRNA) with aspartate to facilitate protein translation, and its mutations are thought to lead to non-synonymous amino acid substitutions that impair aminoacylation or destabilize enzyme structure [120]. Although the genetic mutations associated with HBSL are known, the specific pathological mechanisms are unknown.

Studies using patient-specific induced pluripotent stem cells (iPSCs) have advanced our understanding by allowing researchers to generate 3D brain organoids that recapitulate human development in a way rodent models cannot [121]. These organoids can be enriched for functional oligodendrocytes and neurons, providing a platform to perform bulk and single-cell transcriptomics to pinpoint early cellular dysregulation [122]. By using reporter lines-such as those tracking the SOX10 gene-scientists can monitor the specification and maturation of oligodendroglial cells in real-time, making it possible to identify exactly where the developmental process fails in patients with specific genetic variants [122]. This high-throughput approach serves as a valuable tool for functional neurogenomics and screening for novel therapeutics [121].

Furthermore, iPSC-derived model systems are increasingly used to study cognitive and functional properties by analyzing synchronized neural activity and the formation of "learnable" neural networks. By evaluating features such as short-term plasticity and the balance of excitatory and inhibitory synaptic transmission, researchers can gain insights into how molecular defects translate into the functional and cognitive impairments observed in complex neurological disorders.

### Xenotransplantation of human neurons in animal brains

Human-rodent chimeric models provide a powerful platform to study human-specific neural development and disease states within the physiological context of a living brain [123]. Experiments typically involve differentiating human pluripotent stem cells (PSCs) or induced pluripotent stem cells (iPSCs) into neural progenitor cells (NPCs), cortical neurons, or 3D-organized organoids. These human cells are then xenotransplanted into a rodent brain, targeting the lateral ventricles, cerebral cortex, or specific injury sites, often at neonatal or even embryonic stages in utero, allowing them to integrate into host circuits [124, 125]. These cells may be grafted into healthy mice as well as transgenic models of Alzheimer’s disease (AD), and ischemic stroke injuries to recapitulate human-specific pathological development or to study neural circuit restoration [126, 127]. In the chimeric brain, the human neurons can be tracked using fluorescent proteins; advanced techniques such as longitudinal two-photo imaging, optogenetics, ex vivo whole-cell patch-clamp recordings, and single-nucleus RNA sequencing have been employed to monitor the structural maturation, synaptic plasticity, and transcriptomic signatures of the grafted cells over several months [124, 126, 127].

This model can address critical questions regarding human-specific vulnerabilities to disease, such as why human neurons undergo robust neurodegeneration and necroptosis when exposed to amyloid-beta plaques while mouse neurons remain resilient [126]. Furthermore, the model allows for the investigation of graft-host crosstalk, demonstrating how human neurons can integrate functionally to respond to sensory stimuli or drive recovery after injury through mechanisms like enhanced angiogenesis and synaptic restorations [127]. By bypassing the limitations of in vitro systems, these chimeras offer unique insights into how the host environment influences synaptogenesis and whether specific genetic mutations, such as those in Timothy syndrome or Frontotemporal dementia, manifest differently in an in vivo setting [128]. At the meeting, xenotransplantation of human neural stem cells into mouse brains followed by prolonged stress of animals was presented. This approach provides an individualized model to elucidate the neurobiological consequences of stress and evaluate regenerative therapies. The findings open new avenues to explore mechanisms that render neural grafts vulnerable to stress and highlight advances in molecular imaging, electrophysiological monitoring, and transcriptomic profiling as promising tools for further investigation.

It remains to be confirmed that chronic stress has pervasive, impactful modulatory effects on neuronal plasticity, synaptic remodeling, and neuroimmune interactions, potentially threatening the viability and functional integration of transplanted human neurons into xenograft models. It is yet to be tested whether stress-related glucocorticoids, including cortisol, affects synaptic plasticity in the host environment, potentially reducing the survival, differentiation, and functional connectivity of transplanted neurons.

This exciting new avenue could lead to more effective use of xenotransplantation techniques in neuro-regenerative medicine, including the use of personalized stem cell-derived neuronal constructs and genetic modifications to render donor neurons stress-resistant. Additional strategies could involve capacity-driven perdurable neuromodulation and integration, particularly in the context of neurodegenerative disorders and psychiatric conditions driven by stress.

## Application of RDoC framework in cognitive and psychiatric disorders

The Research Domain Criteria (RDoC) is a paradigm shift in psychiatry/cognitive neuroscience that describes a multi-dimensional framework across human function in which specific behavioral outcomes are linked to neural circuits and molecular mechanisms [129].

Discussions noted the importance of integrating genetics, neuroimaging, and computational modeling across studies to elucidate the biological basis of mental disorders. Researchers emphasized how RDoC lends itself to a cross-domain approach in which dimensions including cognitive control, reward processing, and emotional regulation are studied in their functional circuits rather than their diagnostic categories [130]. This framework facilitates a data-driven conceptualization of psychiatric syndromes and integrates biomarker discovery, psychophysiological metrics, and digital phenotyping to enhance diagnostic accuracy and therapeutic delivery. Technologies such as multi-omic profiling (multi-OMICs), real-time neural recording, and machine learning (ML) analysis of behavior, discussed at the meeting, further illustrate how RDoC can be applied to refine personalized medicine in psychiatry [131]. Treatment response predictions in schizophrenia, depression, and autism spectrum disorder from pathways derived from neurocognitive markers and circuit-specific dysfunctions are determined by RDoC [130, 132–134]. Furthermore, the meeting discussed the utility of machine learning algorithms to mine the large-scale RDoC datasets to classify individuals by neurofunctional patterns rather than clinical labels [135].

Further research combining connectomics, epigenetic profiling, and neuromodulatory interventions could crystallize the RDoC model, providing a better and more biologically valid framework for understanding and treating neuropsychiatric disorders. This has been proposed in a Brain-, Spine-, and Mental-Health Screening Methodology (NEUROSCREEN) for Healthcare Systems in a recent report [136].

The definitions and domains of the RDoC (http://www.nimh.nih.gov/research-funding/rdoc) framework helps bridge between molecular mechanisms and clinical neurodiagnostics and therapeutics that advance a precision neurocognitive medicine approach [137, 138]. The MCCS-NYUAD meeting perspectives push us toward dimensional rather than categorical delimitations of cognitive dysfunctions, and that is crucial for translational progress. Table 1 demonstrates an RDoC-Informed matrix for molecular cognition and its units of analysis. In such a framework, Molecular regulators ranging from HDACs, HATs, m6A methyltransferases, lncRNAs, microRNAs, to neuroimmune mediators determine cognitive function. These modulate the activity of specific brain circuits, such as the CA2-CA1 hippocampal pathways for memory, anterior cingulate cortex involved in spatial encoding, and insular cortex for immune-cognitive integration. These effects are carried out behaviorally, with deficits in reward-aversion, spatial decisions, and social memory. These links are further confirmed by neuroimaging and electrophysiological resting-state and task-based patterns of brain activity (Table 1).

**Table 1:** An RDoC-Informed Matrix for Molecular Cognition and its Units of Analysis

Domain	Construct	Unit of analysis	Examples from MCCS-NYUAD meeting
Cognitive systems	Working memory	Molecules/Genes	The epigenetic regulators (such as histone acetylation, DNA methylation and lncRNAs) regulate the hippocampal/prefrontal circuits of the memory
		Cells/Circuits	Synaptic tagging and capture in hippocampal area CA2 through CA1 circuits; metaplasticity in the CA1 enabling memory flexibility
		Behavior/Self-Report	Memory retrieval and forgetting by synaptic remodeling (e.g., engram synapse labeling tracing, episodic gist abstraction)
		Physiology	Spontaneous DMN oscillations are correlated with cognitive flexibility and implicit learning (idling brain hypothesis)
Systems for social processes	Social communication	Genes/Molecules	Oxytocin and vasopressin receptor activity controlling social memory synapses in CA2 and mPFC
		Circuits	CA2-CA1 and VTA-hippocampus projections mediating social recognition memory
		Behavior	Social memory deficit in perineuronal net-deficient CA2
Negative valence systems	Loss and frustrative nonreward	Molecules/Synapses	Dopaminergic regulation of ventral hippocampal activity during negative affective states and rewarded behaviors m6A RNA methylation is critical for memory formation
		Physiology	Chronic social stress-induced synaptic depression affecting reward versus aversion integration
Arousal/Regulatory systems	Circadian rhythms and sleep	Circuits/Neurochemicals	SCN-HPA axis dysfunction: memory consolidation impaired by cortisol and melatonin rhythms
		Molecular Profiles	SCN and habenula specific stress-mediated changes in histone and DNA methylation driving resilience
Sensorimotor systems	Motor function	Neurophysiology	Cerebellar metaplasticity and motor control by perineuronal nets
		Imaging/Connectivity	fMRI/DTI-based evidence of post-cataract surgery white matter plasticity, motor pathway remyelination
Positive valence systems	Reward learning	Cells/Circuits	VTA-hippocampus-amygdala triad in learning and memory; synaptic LTP through dopamine gating
		Molecular Dynamics	Epigenetic and synaptic control of excitatory synapses in the mouse neocortex with a reduction of excitatory synapses
Neurodevelopmental factors	Neuroplasticity and Myelination	Genes/Developmental Genes	Oligodendrocyte dysfunction, hypomyelination, and cytokines inhibiting remyelination
		Imaging	DTI demonstrates loss of myelin integrity in corticospinal and cerebellar tracts

Each domain has implications that address cognitive, behavioral, motor, and sensory processing, which serve as a basis for precision diagnostics (including AI-driven neurodiagnostics) and targeted interventions including neuroimmune modulation strategies.

**Fig. 2:**
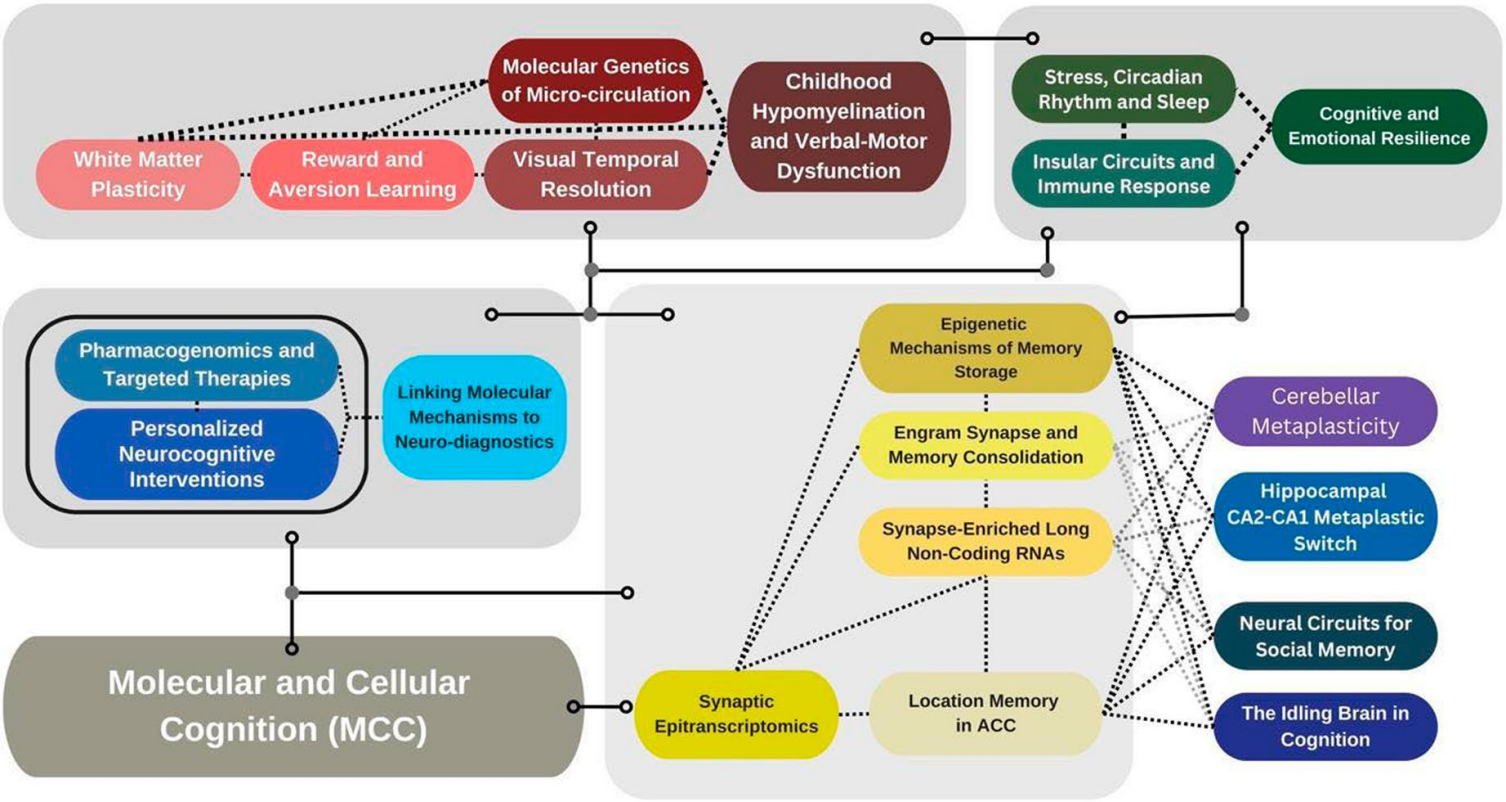
Molecular-to-Clinical Trajectory Concept Map. This framework illustrates how the discussions at MCCS-NYUAD align the molecular and cellular foundations of cognition with systems-level brain plasticity and clinical translation. It integrates key domains of Molecular and Cellular Cognition, including synaptic epitranscriptomics, engram consolidation, and memory-related epigenetic regulation, with circuit-level metaplasticity across hippocampal and cerebellar networks. Together, these mechanisms shape cognition, behavior, and resilience. By linking molecular discoveries to neuroimmune modulation, stress adaptation, and sensory-motor integration, the model supports precision diagnostics and targeted therapeutic strategies for neurodevelopmental and neurodegenerative disorders

Concept map: the molecular-to-clinical trajectory (Fig. 2).

### Concluding remarks: open questions, emerging technologies, and priority research areas

The MCCS-NYUAD meeting convened researchers, clinicians, and students around the globe to discuss recent advancements in topics spanning from molecules to cognition, and to diseases. The conversations have inspired us to work toward bridging scientific discoveries with clinical translations of precision diagnostics and personalized interventions, providing mechanistic pathways that modulate cognition and behavior in healthy and diseased states.

New questions have been raised. How do synaptic epitranscriptomics regulate short- or long-term memory, or cognitive flexibility? Can metaplasticity serve as a therapeutic target for cognitive resilience? How does chronic stress reprogram neural epigenetics and gene expression underlying circuit plasticity? Can we identify individual-specific molecular patterns (e.g., transcriptomic, epigenetic, proteomic) associated with cognitive traits such as memory strength, attention, or cognitive flexibility? Can we track dynamic molecular plasticity during cognitive transitions or treatment responses? Can engram-specific molecular profiling reveal targets for cognitive intervention?

Addressing these questions requires the development of new technologies and tools, including optogenetics; bulk and single-cell transcriptomics at isoform level; real-time ribosome profiling; molecular imaging; CRISPR-based epigenetic editing; and pharmacological HDAC inhibition. On the clinical side, advances in neuromodulatory interventions and pharmacogenomics, together with mnemonic agents used in non-invasive brain stimulation (TMS, tDCS, etc.) are playing an increasingly important role. These emerging technologies have the potential to recalibrate metaplasticity dynamics, thereby promoting more favorable learning and memory outcomes. RNA-targeted based therapies also represent exciting avenues for reversing stress-induced epigenetic dysregulation; however, each intervention will require careful assessment of long-term stability and specificity. Ongoing studies will further determine whether non-invasive neuromodulatory techniques, including vagal nerve stimulations (VNS), transcranial direct current stimulations (tDCS), and neuroimmune pharmacotherapies, may ameliorate cognitive decline secondary to inflammation.

Finally, we highlight the promise of AI-based technologies to transform precision neurodiagnosis and personalized neuromedicine by integrating large-scale genomic, epigenetic, and metabolomic data to predict cognitive decline, treatment response, and disease progression. AI-augmented clinical decision support systems that combine neuroimaging, genetic risk profiles, and molecular diagnostics will enable targeted neuroprotective strategies in cognitive impairment.

## Data Availability

Data sharing not applicable to this article as no datasets were generated or analyzed during the current report.

## Abbreviations

AAV: Adeno-associated virus
ACC: Anterior cingulate cortex
AD: Alzheimer’s disease
AI: Artificial intelligence
aIC: Anterior insular cortex
AM: Amplitude modulation
ANS: Autonomic nervous system
APC: Adenomatous polyposis coli (RNA-binding protein involved in axonal transport)
AspRS: Aspartyl transfer RNA synthetase
BLA: Basolateral amygdala
CA1: Cornu ammonis area 1 of the hippocampus
CA2: Cornu ammonis area 2 of the hippocampus
CA3: Cornu ammonis area 3 of the hippocampus
CIR: Conditioned immune response
CRISPR: Clustered regularly interspaced short palindromic repeats
DG: Dentate gyrus
DMN: Default mode network
DNA: Deoxyribonucleic acid
DRN: Dorsal raphe nucleus
DTI: Diffusion tensor imaging
EEG: Electroencephalography
E/I: Excitatory to inhibitory balance
ERK: Extracellular signal regulated kinase
FDG-PET: Fluorodeoxyglucose positron emission tomography
fMRI: Functional magnetic resonance imaging
GABA: Gamma aminobutyric acid
HATs: Histone acetyltransferases
HBSL: Hypomyelination with brainstem and spinal cord involvement and leg spasticity
HDAC: Histone deacetylase
IC: Insular cortex
iPSC: Induced pluripotent stem cell
LEC: Lateral entorhinal cortex
LHb: Lateral habenula
lncRNA: Long non-coding RNA
LTP: Long term potentiation
m6A: N6 methyladenosine
MCC: Molecular and cellular cognition
MCCS: Molecular and cellular cognition society
MEG: Magnetoencephalography
ML: Machine learning
mPFC: Medial prefrontal cortex
NMDA: N methyl D aspartate (receptor)
NPC: Neural progenitor cell
OCD: Obsessive compulsive disorder
OMIM: Online mendelian inheritance in man
P4: Predictive, preventive, precision, and participatory medicine model
PET: Positron emission tomography
pIC: Posterior insular cortex
PSC: Pluripotent stem cell
PTSD: Post traumatic stress disorder
REM: Rapid eye movement sleep
RNA: Ribonucleic acid
RDoC: Research domain criteria
rs-fMRI: Resting state functional magnetic resonance imaging
SCN: Suprachiasmatic nucleus
SegInt: Segregation integration task
SWS: Slow wave sleep
tACS: Transcranial alternating current stimulation
tDCS: Transcranial direct current stimulation
TFF: Two flash fusion task
TMS: Transcranial magnetic stimulation
V1/V2/V5(MT): Primary, secondary, and middle temporal visual cortices
VNS: Vagus nerve stimulation
VTA: Ventral tegmental area
